# A phase II study of raltitrexed and gemcitabine in patients with advanced pancreatic carcinoma

**DOI:** 10.1038/sj.bjc.6602368

**Published:** 2005-01-25

**Authors:** J J Arends, H P Sleeboom, M B L Leys, D ten Bokkel Huinink, R S de Jong, J M Smit, J W R Nortier, M E T Tesselaar

**Affiliations:** 1 Department of Clinical Oncology, Leiden University Medical Center, Albinusdreef 2, 2333 ZA Leiden, The Netherlands; 2 Departments of Internal Medicine, Leyenburg Hospital, PO Box 40551, 2504 LN The Hague, The Netherlands; 3 Medical Center Rijnmond, PO Box 9119, 3007 AC Rotterdam, The Netherlands; 4 Diakonessen Hospital, PO Box 80250 3508 TG Utrecht, The Netherlands; 5 Martini Hospital, PO Box 30033. 9700 RM Groningen; 6 Gelre Hospital, PO Box 9014, 7300 DS Apeldoorn, The Netherlands

**Keywords:** gemcitabine, pancreatic cancer, raltitrexed, phase II

## Abstract

Advanced adenocarcinoma of the pancreas has a very poor prognosis. The aim of this study was to assess the efficacy and tolerability of a combination of the chemotherapeutic agents gemcitabine and raltitrexed. Chemonaïve patients with advanced adenocarcinoma of the pancreas were treated with a combination of raltitrexed (3.5 mg m^−2^ on day 1 of a 21-day treatment cycle) and gemcitabine (800 mg m^−2^ intravenously (i.v.) on days 1 and 8 of a 21-day cycle). Between April 2000 and February 2003, 27 patients were enrolled onto the study. The mean duration of treatment was 11 weeks. Four of 27 patients experienced at least one episode of grade 3 or 4 neutropenia. One patient with grade 4 neutropenia died due to sepsis. Four of 27 patients experienced grade 4 diarrhoea. There was one partial remission (4%) and 12 patients experienced disease stabilisation (44%). The 6-month and 1-year survival rates were 37 and 11%, respectively. Symptomatic benefit occurred in seven (26%) patients. We conclude that a combination of raltitrexed and gemcitabine, using the schedule and doses in this study, cannot be recommended for patients with advanced pancreatic cancer.

Adenocarcinoma of the pancreas is a disease that rapidly leads to fatality, which causes annually over 40 000 deaths in Europe (Pisani *et al*, 1999) and more than 210 000 world wide (Parkin *et al*, 2001). The median survival of untreated patients is 3 months (Palmer *et al*, 1994; Glimelius *et al*, 1996). A median survival of up to 14.3 months can be achieved with radical surgery, but this is possible in less than 20% of patients, as pancreatic carcinoma tends to present late in its course (Conlon *et al*, 1996; Haycox *et al*, 1998).

All other patients, with unresectable disease, are potential candidates for systemic treatment, although unfortunately, pancreatic carcinomas are rather chemoinsensitive (Kornmann *et al*, 1999). In a study of patients with advanced pancreatic cancer by Burris *et al*, gemcitabine provided clinical benefit to 24% of patients compared to 5% with 5-fluoruracil (5-FU), and the survival rate at 12 months was 18% for gemcitibine patients compared to 2% for 5-FU patients (Burris *et al*, 1997). No single agent or combination of agents has been proven superior to gemcitabine alone, and although the objective response is low (below 10%), gemcitabine is the drug of choice for patients with advanced pancreatic cancer (Burris *et al*, 1997; Shore *et al*, 2003).

Recent advances in cancer genetics have led to the development of novel rationally designed chemotherapeutic agents. A promising target for pancreatic cancer therapy is the K-Ras oncogene product and its signalling pathway. K-Ras mutations that encode for activated proteins are found in more than 90% of all pancreatic cancers (Jaffee *et al*, 2002; Shore *et al*, 2003). In order to bind to the cell membrane, Ras protein has an additional farnesyl group. Raltitrexed (Tomudex®; AstraZeneca Pharmaceuticals, Holland) is a quinazoline folate analogue, which acts as a pure and specific thymidylate synthetase (TS) inhibitor, designed to inhibit K-ras protein farnesylation. *In vitro* single dosing has shown a similar duration of inhibition of TS as bolus 5-FU (Ford *et al*, 2002). Large randomised studies of raltitrexed have demonstrated equivalent response rates and reduced toxicity compared to the combination of 5-FU and leucovorin in colorectal cancer (Cunningham, 1998). A phase II study with raltitrexed in patients with pretreated pancreatic cancer showed a response rate of 5% (Pazdur *et al*, 1995).

Owing to some single agent efficacy, a nonoverlapping toxicity profile and a difference in mechanism of action, raltitrexed is an interesting agent to combine with gemcitabine. In order to assess the efficacy and tolerability of the combination of raltitrexed and gemcitabine in patients with nonresectable advanced pancreatic cancer, a multicentre phase II trial was started in April 2000.

## PATIENTS AND METHODS

### Study design objectives

The primary objective of this phase II study was to determine the efficacy of a combination of gemcitabine and raltitrexed in advanced or metastatic adenocarcinoma of the pancreas. Secondary objectives were to determine the duration of objective response and to evaluate the toxicity profile of this treatment.

### Patient eligibility

Patients with inoperable advanced or metastatic adenocarcinoma of the pancreas had to be chemonaïve. Further inclusion criteria consisted of histologically or cytologically proven pancreatic adenocarcinoma, a World Health Organization (WHO) status of two or less and the presence of a two-dimensional measurable lesion of at least 1 cm. Patients had to be older than 18 years with an estimated life expectancy of at least 8 weeks. Adequate organ function was defined as: a haemoglobin level ⩾6.5 mmol l^−1^, a white blood cell count ⩾4000 *μ*l^−1^, a platelet count ⩾100 000 *μ*l^−1^, a creatinine clearance of ⩾65 ml min^−1^ (calculated according to the formula of Cockcroft and Gault, 1976), bilirubin levels ⩽3 times the upper normal laboratory value, serum transaminase levels <2.5 times the normal laboratory values without known liver involvement or <5 times the normal laboratory values with known liver involvement. Patients who had been treated with radiotherapy for pancreatic cancer, and patients with symptomatic cerebral or leptomeningeal metastases were excluded. The local ethics committees approved this study and written informed consent was obtained from all patients before entry to the study.

### Treatment protocol

Raltitrexed (Tomudex®) was administered intravenously (i.v.) at a dose of 3.5 mg m^−2^ over 15 min on day 1 of a 21-day treatment cycle. Gemcitabine (Gemzar®; Eli Lilly, Indianapolis, USA) was administered at a dose of 800 mg m^−2^ i.v. over 30 min on days 1 and 8 of a 21-day cycle. Courses were administered every 3 weeks, as long as patients benefited, and there was no evidence of progressive disease (PD).

Adverse reactions were evaluated according to Common Toxicity Criteria of the National Cancer Institute of Canada (NCI – CTC). In the event of clinically relevant toxicity during the first cycle of treatment, dosing was delayed until all signs of toxicity had resolved or at least improved. Any patient who required more than 14 days for recovery was removed from the study. For all further cycles, administered doses were based on toxic effects observed during the previous cycle. In the case of grade 3 or 4 haematological toxicity requiring more than 1 week delay of the next treatment cycle, or febrile neutropenia, both drug doses were reduced by 25% for subsequent cycles. The raltitrexed dose was reduced to 75 or 50%, respectively, if grade 3 or 4 nonhaematological toxicity occurred during the previous treatment cycle. Doses of both chemotherapeutic agents were reduced in the case of grade 2 or 3 diarrhoea and discontinued in the case of grade 4 diarrhoea. In the event of renal function impairment, raltitrexed was reduced to 75 or 50%, if creatinine clearance was 55–65 or 25–54 ml min^−1^, respectively. In both cases cycle duration was extended to 28 days. When creatinine clearance was less than 25 ml min^−1^, raltitrexed treatment was terminated. In the case of grade 3 or 4 toxicity after a dose reduction, patients were removed from the study.

### Clinical evaluation

Toxicity analyses were applied to patients who received at least one course of chemotherapy, while response evaluation was assessed after every two cycles of chemotherapy, either using a CT scan or clinically according to WHO standard criteria (Miller *et al*, 1981). Subjective symptoms (pain score, analgesic use and performance status), serious adverse reactions, physical examination, performance status and other clinical benefit response parameters were recorded before each treatment cycle.

### Statistics

According to the optimal two-stage design, for a target activity level of at least 20% response rate, two objective responses had to be observed in the first 21 assessable patients (alpha and beta error probabilities 0.005 and 0.01, respectively), otherwise this trial would be terminated. Time-related parameters were analysed using Kaplan–Meier on an intention-to-treat analysis.

## RESULTS

Between April 2000 and February 2003, 27 patients from six institutions were enrolled onto the study. All patients were eligible. The main clinical characteristics at study entry are listed in Table 1. The median age was 59 years (range 42–74 years) and the performance status was 44, 44 and 11% for WHO 0, 1 and 2, respectively. No patients had received radiotherapy or chemotherapy prior to the study. In all, 16 (59%) patients had metastatic disease at the time of registration. A total of 95 cycles were administered (mean four cycles per patients, range 1–14). The mean duration of treatment was 11 weeks.

Six patients (22%) did not start with the second course of chemotherapy due to objective or subjective toxicity and were not assessable for efficacy (Table 2). One (4%) patient showed a partial response (95% CI-3–11%). A total of 12 (44%) patients showed disease stabilisation or a minor response with a median duration of 3 months (range 2–7 months). In eight patients (30%), the tumour was progressive despite treatment. The overall median survival was 5.5 months (range 1–16 months). The median time to progression was 3.4 months (range 0–12 months). At the time of study evaluation, two patients were still alive at 99 and 106 days of follow-up. The 6-month and 1-year survival was 37 and 11%, respectively.

The toxicity for up to six treatment cycles was evaluated for all patients and results are summarised in Table 3. Four patients were unwilling to undergo further treatment because of subjective toxicity. Haematological toxicities were grades 3–4 in four patients with leucopoenia and in three patients with thrombocytopenia. One patient with grade 4 leucopoenia died due to septicaemia. Elevated transaminases and alkaline phosphatase were frequently observed in the patients. However, these abnormalities were self-limiting. In eight patients, transaminases were elevated before entry to the study and were therefore most probably disease related. Grade 1–2 nausea occurred in 41% of the treatment cycles. Grade 1–3 diarrhoea occurred in 17% of cycles, leading to hospitalisation in four (15%) patients. In two patients, with grade 2 and 3 diarrhoea, respectively, the doses of gemcitabine and ralitrexed were not reduced according to the protocol. Both patients experienced grade 4 diarrhoea in the following treatment cycle. No dose modifications for renal impairment needed to be performed over the first six treatment cycles.

Five of the 27 patients who reported pain at study entry reported less pain after treatment and two patients experienced weight gain. Therefore, seven patients (26%) were classified as clinical benefit responders. Four patients (15%) were stable in both primary (ie pain intensity and WHO performance status) and secondary (weight) parameters.

## DISCUSSION

The effects of 5-FU in pancreatic carcinoma have been extensively studied and reported response rates for single agent 5-FU treatment in patients with pancreatic cancer range from 0 to 19% (Warshaw and Fernandezdelcastillo, 1992; Haller, 2003). Although gemcitabine is generally approved as a first-line treatment for advanced pancreatic cancer, the objective responses are low and the median survival benefit is modest in comparison with 5-FU alone (Burris *et al*, 1997). Owing to disappointing results from chemotherapy in pancreatic cancer, combination regimens have now been developed. Combinations of 5-FU-like drugs and gemcitabine show synergistic antitumour activity *in vitro* (Ren *et al*, 1998; Peters *et al*, 2000). In patients with advanced cancer, it has been shown that gemcitabine increases systemic 5-FU exposure (Correale *et al*, 2003a), and several studies have tested the combination of these two drugs in pancreatic cancer with response rates of up to 31% and median survival rates of up to more than 1 year (Correale *et al*, 2003b; Lee *et al*, 2004). One randomised phase III study with gemcitabine in combination with 5-FU *vs* gemcitabine alone did not demonstrate any improvement in median survival (Berlin *et al*, 2002). However, 5-FU was given as a bolus infusion and there is evidence that continuous infusion might be superior to bolus administrations (Hansen *et al*, 1988; Aschele *et al*, 1992; Hidalgo *et al*, 1999). Thus, the method of administration may have influenced the outcome of the study by Berlin *et al* and so the combination of these two drugs remained attractive.

A full paper was published on one study by Kralidis *et al* (2003) involving the combination of raltitrexed and gemcitabine in patients with locally advanced and metastatic pancreatic cancer. In this study, the patients were treated with raltitrexed (3 mg m^−2^ in 15 min infusion) on day 1 and gemcitabine (1000 mg m^−2^) on days 1 and 8. In comparison to our study, patients had worse prognostic factors (Cubiella *et al*, 1999), that is, 20% of patients had a WHO score of 0 compared to 44% in our study and 84% of patients had metastatic disease compared to 59% in our study. The reported 1-year survival (11%) and tumour growth stabilisation rate (overall response+stable disease) are more or less equal in both studies, but the reported response rates differ (12% compared to 4% in our study). We used a relatively high dose of bolus raltitrexed in combination with a lower dose of gemcitabine. Laboratory research on the effects of raltitrexed on elevation of plasma 2′ deoxyuridine as a marker for TS inhibition suggests that there is a plasma drug level above which no further TS inhibition is accomplished (Ford *et al*, 2002). Thus, high plasma concentrations of raltitrexed after bolus infusion might be responsible for the increased amount of gastrointestinal toxicity in our patients, without an increase in therapeutic effect. In our study, seven patients stopped further chemotherapy due to subjective or objective toxicity. One of these patients had a potential partial response, but this could not be confirmed objectively. The difference observed between these studies may also be caused by the relatively low dose of gemcitabine administered to our patients. Preclinical data using human tumour cell lines indicated a possible dose–response relationship, suggesting that exposure to a higher concentration of gemcitabine might correlate with improved clinical effectiveness (Von Hoff, 1996).

The reported 1-year survival and clinical benefit ratios in our study and the study of Kradalis *et al* are not better than the results reported with gemcitabine as a single agent (Burris *et al*, 1997). One reason for this could be related to the design of both studies. Preclinical data suggest that gemcitabine given before 5-FU produces more synergistic antitumour activity in pancreas carcinoma cell lines (Correale *et al*, 2000). In our study and the study of Kradalis *et al*, gemcitabine was administered after the raltitrexed. In our study, gemcitabine was administered as a 30 min infusion and not as a fixed dose rate (FDR). In a randomised phase II study by Tempero *et al* (2003) comparing gemcitabine treatment as a standard 30 min infusion to administration at an FDR of 10 mg m^−2^ min^−1^ over 150 min, the 1- to 2- and even 3-year survival rates were in favour of the FDR. This was also seen in a phase II study in which patients with advanced pancreatic cancer given FDR gemcitabine in combination with an oral fluoropyrimidine had a median survival of almost 1 year (Feliu *et al*, 2002).

We conclude that this combination of raltitrexed and gemcitabine given in a sequential schedule has a moderate activity. There is no evidence suggesting that this combination is advantageous over gemcitabine or 5-FU alone in the treatment of advanced pancreatic cancer.

## Figures and Tables

**Table 1:** Patient characteristics

**Characteristic**	***n***
Number of patients	27
*Age (years)*	
Median	59
Range	42–74
	
Female/male	13/14
*Disease at presentation*	
Locally advanced	11
Metastatic	16
	
*WHO performance status*	
0	12
1	12
2	3
	
*WHO pain intensity*	
0	6
1	6
2	6
3	8
4	1
	
Weight loss >5% pretreatment	13

WHO=World Health Organization.

**Table 2:** Overall objective response

	**No. of patients (*n*=27)**	**%**
PR	1	4
SD	12	44
PD	8	30
Not assessable	6	22

PR=partial response; SD=stable disease; PD=progressive disease.

**Table 3:** Toxicity

	**WHO grade**			
**Toxicity**	**1**	**2**	**3**	**4**
Anaemia	9 (19)	4 (5)	—	1 (1)
Leucopoenia	3 (9)	4 (6)	0 (1)	4 (4)
Thrombocytopenia	0 (2)	—	3 (3)	—
Elevated ASAT	10 (30)	5 (7)	4 (5)	1 (1)
Elevated ALAT	9 (23)	6 (12)	8 (10)	—
Elevated alkaline phosphatase	7 (23)	9 (12)	2 (2)	—
Elevated bilirubin	2 (2)	1 (1)	2 (2)	4 (4)
Nausea	8 (29)	7 (12)	7 (7)	—
Vomiting	9 (16)	8 (10)	2 (2)	—
Diarrhoea	6 (8)	0 (2)	2 (3)	4 (4)
Mucositis	4 (5)	—	2 (2)	—
Cutaneous	2 (6)	2 (3)	—	—
Flu-like symptoms	4 (9)	5 (7)	—	—

ASAT=serum glutamic oxaloacetic transaminase; ALAT=serum glutamic pyruvic transaminase; WHO=World Health Organization.

## References

[bib1] Aschele C, Sobrero A, Faderan MA, Bertino JR (1992) Novel mechanism(s) of resistance to 5-fluorouracil in human colon cancer (Hct-8) sublines following exposure to 2 different clinically relevant dose schedules. Cancer Res 52: 1855–1864 1532346

[bib2] Berlin JD, Catalano P, Thomas JP, Kugler JW, Haller DG, Benson AB (2002) Phase III study of gemcitabine in combination with fluorouracil *versus* gemcitabine alone in patients with advanced pancreatic carcinoma: Eastern Cooperative Oncology Group Trial E2297. J Clin Oncol 20: 3270–3275 12149301 10.1200/JCO.2002.11.149

[bib3] Burris HA, Moore MJ, Andersen J, Green MR, Rothenberg ML, Madiano MR, Cripps MC, Portenoy RK, Storniolo AM, Tarassoff P, Nelson R, Dorr FA, Stephens CD, VanHoff DD (1997) Improvements in survival and clinical benefit with gemcitabine as first-line therapy for patients with advanced pancreas cancer: a randomized trial. J Clin Oncol 15: 2403–2413 9196156 10.1200/JCO.1997.15.6.2403

[bib4] Cockcroft DW, Gault MH (1976) Prediction of creatinine clearance from serum creatinine. Nephron 16: 31–41 1244564 10.1159/000180580

[bib5] Conlon KC, Klimstra DS, Brennan MF (1996) Long-term survival after curative resection for pancreatic ductal adenocarcinoma – clinicopathologic analysis of 5-year survivors. Ann Surg 223: 273–279 8604907 10.1097/00000658-199603000-00007 PMC1235115

[bib6] Correale P, Cerretani D, Marsili S, Pozzessere D, Petrioli R, Messinese S, Sabatino M, Roviello F, Pinto E, Francini G, Giorgi G (2003a) Gemcitabine increases systemic 5-fluorouracil exposure in advanced cancer patients. Eur J Cancer 39: 1547–1551 12855261 10.1016/s0959-8049(03)00361-7

[bib7] Correale P, Cerretani D, Petrioli R, Marsili S, Giorgi G, Francini G (2000) Gemcitabine and 5 fluorouracil in the treatment of patients with pancreas and colo-rectal carcinoma: a clinical and pharmacological study. ICACT 10th International Congress on Anti-Cancer Treatment January 31–February 3 2000, Paris, France, abs. no. 1–293

[bib8] Correale P, Messinese S, Marsili S, Ceciarini F, Pozzessere D, Petrioli R, Sabatino M, Cerretani D, Pellegrini M, Di Palma T, Neri A, Calvanese A, Pinto E, Giorgi G, Francini G (2003b) A novel biweekly pancreatic cancer treatment schedule with gemcitabine, 5-fluorouracil and folinic acid. Br J Cancer 89: 239–242 12865908 10.1038/sj.bjc.6601045 PMC2394244

[bib9] Cubiella J, Castells A, Fondevila C, Sans M, Sabater L, Navarro S, Fernandez-Cruz L (1999) Prognostic factors in nonresectable pancreatic adenocarcinoma: a rationale to design therapeutic trials. Am J Gastroenterol 94: 1271–1278 10235206 10.1111/j.1572-0241.1999.01018.x

[bib10] Cunningham D (1998) Mature results from three large controlled studies with raltitrexed (‘Tomudex’). Br J Cancer 77: 15–21 10.1038/bjc.1998.421 PMC2149726 9579851

[bib11] Feliu J, Mel R, Borrega P, Gomez LL, Escudero P, Dorta J, Castro J, Vazquez-Estevez SE, Bolanos M, Espinosa E, Baron MG (2002) Phase II study of a fixed dose-rate infusion of gemcitabine associated with uracil/tegafur in advanced carcinoma of the pancreas. Ann Oncol 13: 1756–1762 12419748 10.1093/annonc/mdf286

[bib12] Ford HER, Mitchell F, Cunningham D, Farrugia DC, Hill ME, Rees C, Calvert AH, Judson IR, Jackman AL (2002) Patterns of elevation of plasma 2′-deoxyuridine, a surrogate marker of thymidylate synthase (TS) inhibition, after administration of two different schedules of 5-fluorouracil and the specific TS inhibitors raltitrexed (Tomudex) and ZD9331. Clin Cancer Res 8: 103–109 11801545

[bib13] Glimelius B, Hoffman K, Sjoden PO, Jacobsson G, Sellstrom H, Enander LK, Linne T, Svensson C (1996) Chemotherapy improves survival and quality of life in advanced pancreatic and biliary cancer. Ann Oncol 7: 593–600 8879373 10.1093/oxfordjournals.annonc.a010676

[bib14] Haller DG (2003) Chemotherapy for advanced pancreatic cancer. Int J Radiat Oncol Biol Phys 56: 16–23 12826247 10.1016/s0360-3016(03)00448-6

[bib15] Hansen R, Quebbeman E, Ritch P, Chitambar C, Anderson T (1988) Continuous 5-fluorouracil (5Fu) infusion in carcinoma of the pancreas – a Phase-II Study. Am J Med Sci 295: 91–93 3344760 10.1097/00000441-198802000-00001

[bib16] Haycox A, Lombard M, Neoptolemos J, Walley T (1998) Review article: current treatment and optimal patient management in pancreatic cancer. Aliment Pharmacol Therap 12: 949–964 9798799 10.1046/j.1365-2036.1998.00390.x

[bib17] Hidalgo M, Castellano D, Paz-Ares L, Gravalos C, Diaz-Puente M, Hitt R, Alonso S, Cortes-Funes H (1999) Phase I–II study of gemcitabine and fluorouracil as a continuous infusion in patients with pancreatic cancer. J Clin Oncol 17: 585–592 10080603 10.1200/JCO.1999.17.2.585

[bib18] Jaffee EM, Hruban RH, Canto M, Kern SE (2002) Focus on pancreas cancer. Cancer Cell 2: 25–28 12150822 10.1016/s1535-6108(02)00093-4

[bib19] Kornmann M, Link KH, Butzer U, Blatter J, Beger HG (1999) *In vitro* concentration response studies of gemcitabine as experimental base for regional chemotherapeutic studies. Eur J Cancer 35: S294

[bib20] Kralidis E, Aebi S, Friess H, Buchler MW, Borner MM (2003) Activity of raltitrexed and gemcitabine in advanced pancreatic cancer. Ann Oncol 14: 574–579 12649104 10.1093/annonc/mdg150

[bib21] Lee J, Park JO, Kim WS, Lee SI, Song SY, Lim dH, Choi SH, Heo JS, Lee KT, Lee JK, Kim K, Jung CW, Im YH, Lee MH, Kang WK, Park K (2004) Phase II study of gemcitabine combined with uracil–tegafur in metastatic pancreatic cancer. Oncology 66: 32–37 15031596 10.1159/000076332

[bib22] Miller AB, Hoogstraten B, Staquet M, Winkler A (1981) Reporting results of cancer treatment. Cancer 47: 207–214 7459811 10.1002/1097-0142(19810101)47:1<207::aid-cncr2820470134>3.0.co;2-6

[bib23] Palmer KR, Kerr M, Knowles G, Cull A, Carter DC, Leonard RCF (1994) Chemotherapy prolongs survival in inoperable pancreatic-carcinoma. Br J Surg 81: 882–885 8044610 10.1002/bjs.1800810629

[bib24] Parkin DM, Bray FI, Devesa SS (2001) Cancer burden in the year 2000. The global picture. Eur J Cancer 37: S4–S66 11602373 10.1016/s0959-8049(01)00267-2

[bib25] Pazdur R, Meropol NJ, Casper ES, Fuchs C, Douglass HO, Vincent M, Abbruzzese JL (1995) Phase II trial of ZD1694 (Tomudex(TM)) in patients with advanced pancreatic cancer. Invest New Drugs 13: 355–358 10.1007/BF00873144 8824356

[bib26] Peters GJ, van der Wilt CL, Van Moorsel CJ, Kroep JR, Bergman AM, Ackland SP (2000) Basis for effective combination cancer chemotherapy with antimetabolites. Pharmacol Ther 87: 227–253 11008002 10.1016/s0163-7258(00)00086-3

[bib27] Pisani P, Parkin DM, Bray F, Ferlay J (1999) Estimates of the worldwide mortality from 25 cancers in 1990. Int J Cancer 83: 18–29 10449602 10.1002/(sici)1097-0215(19990924)83:1<18::aid-ijc5>3.0.co;2-m

[bib28] Ren Q, Kao V, Grem JL (1998) Cytotoxicity and DNA fragmentation associated with sequential gemcitabine and 5-fluoro-2′-deoxyuridine in HT-29 colon cancer cells. Clin Cancer Res 4: 2811–2818 9829747

[bib29] Shore S, Raraty MGT, Ghaneh P, Neoptolemos JP (2003) Chemotherapy for pancreatic cancer. Aliment Pharmacol Therap 18: 1049–1069 14653825 10.1111/j.1365-2036.2003.01781.x

[bib30] Tempero M, Plunkett W, van Haperen VR, Hainsworth J, Hochster H, Lenzi R, Abbruzzese J (2003) Randomized phase II comparison of dose-intense gemcitabine: thirty-minute infusion and fixed dose rate infusion in patients with pancreatic adenocarcinoma. J Clin Oncol 21: 3402–3408 12885837 10.1200/JCO.2003.09.140

[bib31] Von Hoff DD (1996) Activity of gemcitabine in a human tumor cloning assay as a basis for clinical trials with gemcitabine. San Antonio Drug Development Team. Invest New Drugs 14: 265–270 8958181 10.1007/BF00194529

[bib32] Warshaw AL, Fernandezdelcastillo C (1992) Pancreatic-carcinoma. N Engl J Med 326: 455–465 1732772 10.1056/NEJM199202133260706

